# Perpendicular magnetic anisotropy and magnetization dynamics in oxidized CoFeAl films

**DOI:** 10.1038/srep12352

**Published:** 2015-07-20

**Authors:** Di Wu, Zhe Zhang, Le Li, Zongzhi Zhang, H. B. Zhao, J. Wang, B. Ma, Q. Y. Jin

**Affiliations:** 1 Shanghai Engineering Research Center of Ultra-precision Optical Manufacturing , and Key Laboratory of Micro and Nano Photonic Structures (Ministry of Education), Department of Optical Science and Engineering, Fudan University, Shanghai, 200433, China; 2 Department of Physics, Ningbo University, Ningbo, 31500, China

## Abstract

Half-metallic Co-based full-Heusler alloys with perpendicular magnetic anisotropy (PMA), such as Co_2_FeAl in contact with MgO, are receiving increased attention recently due to its full spin polarization for high density memory applications. However, the PMA induced by MgO interface can only be realized for very thin magnetic layers (usually below 1.3 nm), which would have strong adverse effects on the material properties of spin polarization, Gilbert damping parameter, and magnetic stability. In order to solve this issue, we fabricated oxidized Co_50_Fe_25_Al_25_ (CFAO) films with proper thicknesses without employing the MgO layer. The samples show controllable PMA by tuning the oxygen pressure (*P*_O2_) and CFAO thickness (*t*_CFAO_), large perpendicular anisotropy field of ~8.0 kOe can be achieved at *P*_O2_ = 12% for the sample of *t*_CFAO _= 2.1 nm or at *P*_O2 _= 7% for *t*_CFAO _= 2.8 nm. The loss of PMA at thick *t*_CFAO_ or high *P*_O2_ results mainly from the formation of large amount of CoFe oxides, which are superparamagnetic at room temperature but become hard magnetic at low temperatures. The magnetic CFAO films, with strong PMA in a relatively wide thickness range and small intrinsic damping parameter below 0.028, would find great applications in developing advanced spintronic devices.

The ferromagnetic (FM) thin films with perpendicular magnetic anisotropy (PMA) have been widely investigated for practical applications in nanoscale spintronic devices such as spin-transfer-torque magnetic random access memories (STT-MRAMs). The PMA devices were demonstrated to have great advantages over the in-plane ones, including strong thermal stability and low critical switching current density^1^,^2^. Although magnetization switching driven by spin-polarized current has been realized in various PMA systems, e.g. *L*1_0_-ordered FePt thin films^3^,^4^, Co(Fe)/Pt (or Ni) multilayers^2^,^5^,^6^,^7^, amorphous rare earth-transition metal alloys^8^, and CoFeB/MgO systems^9^,^10^,^11^,^12^,^13^,^14^, the advances in high density MRAMs are not so great as expected. Continuing efforts should be made to seek advanced perpendicular thin films showing good performances of high spin polarization *P*, low Gilbert damping parameter *α*, small saturation magnetization *M*_S_, and proper effective perpendicular magnetic anisotropic field *H*_k_^15^.

Recently, as an alternative potential candidate, half-metallic ferromagnetic Co-based full-Heusler alloy film is receiving increased attention due to the full spin polarization. Interfacial PMA has been achieved in Co_2_FeAl (CFA) and Co_2_FeSi Heusler alloy films when they are placed adjacent to the MgO layer, which is believed to be the result of the hybridization between Co- or Fe-3*d* and O-2*p* electron orbitals at the interface of FM/MgO^16^,^17^,^18^,^19^,^20^. In addition, the Gilbert damping parameter for the perpendicularly magnetized CFA sample was reported to be 0.012^18^, much lower than the α value in most PMA structures. However, in spite of the advantages of high spin polarization and low damping parameter in such perpendicular FM/MgO systems, the PMA strength drops dramatically with increasing thickness of the magnetic layer. Usually the magnetization would rotate from out-of-plane to in-plane direction as the magnetic thickness is over ~1.3 nm. Such ultra-thin thickness will not only affect the magnetic stability and spin polarization, but increases the damping constant as well^18^,^21^. As a result, it is of vital importance to seek Co-based heusler alloy films with strong PMA and proper thickness. In this work, without employing the interfacial MgO layer, we have realized PMA in the oxidized CFA (CFAO) thin films with a thickness in the range of 1.0–3.0 nm. The influences of oxidation condition and CFAO thickness (*t*_CFAO_) on magnetic anisotropy will be presented. Moreover, considering that rare work has been performed on the magnetic dynamics for the perpendicular CFA system, we have performed a time-resolved optical pump-probe study. Laser-induced magnetization precession and damping behaviors have been characterized and a damping parameter lower than 0.028 is deduced.

## Results

**Figure 1:**
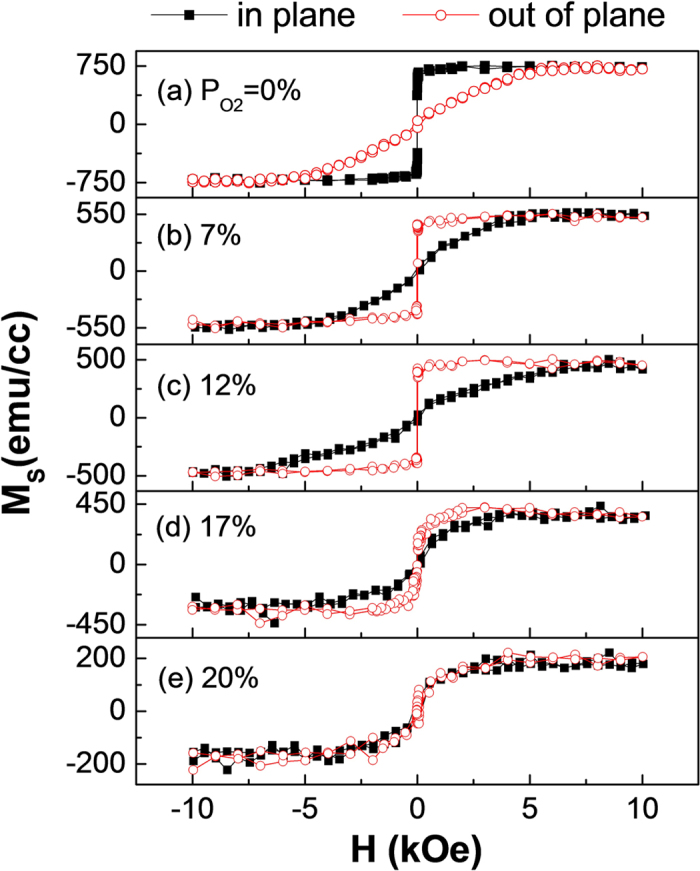
Magnetic hysteresis loops of different oxygen pressure ratios. The in-plane (solid squares) and out-of-plane (open circles) magnetic hysteresis loops for the samples with a structure of Ta(3) /Pd(10) /CFAO(2.1) /Ta(7) fabricated at various oxygen pressure ratios of (**a**) *P*_O2 _= 0%, (**b**) 7%, (**c**) 12%, (**d**) 17%, and (**e**) 20%.

Fig. 1 shows the representative in-plane and out-of-plane magnetic hysteresis loops for Ta/Pd/CFAO/Ta films deposited at various oxygen partial pressure ratios (*P*_O2_). Although the Pd/CoFe interface has been known to favor a perpendicular anisotropy, it is not strong enough to overcome the large demagnetization energy of a thick CFA film. As shown in Fig. 1(a), the CFA layer in a thickness of 2.1 nm has an obvious in-plane easy axis. However, by reactive magnetron sputtering in proper Ar/O_2_ mixtures, the easy axis of CFAO film with the same thickness of 2.1 nm transforms from in-plane to out-of-plane direction, accompanied by a small perpendicular magnetic coercivity (*H*_c^_) less than 20 Oe. Considering that the oxygen atom plays an important role on the observed PMA, we suspect the PMA in our CFAO films may also originate from the interfacial hybridization effect between Co- or Fe-3d and the adjacent O-2p orbitals^22^. However, note that the electronic hybridization of our samples is different from the aligned state in normal FM/oxide structures^23^,^24^, the mechanism for PMA could be different and further study will be performed to deeply understand it. The PMA strength of CFAO is very sensitive to the *P*_O2_. With increasing *P*_O2_ the effective perpendicular anisotropy field *H*_*k*_ increases firstly, showing a maximum value of ~7.8 kOe at *P*_O2 _= 12% and then decreases. Interestingly, further increasing *P*_O2_ up to 20%, no magnetic anisotropy is found. The in-plane and perpendicular hysteresis loops nearly overlap with each other, both with very small remanence and low saturation field, as shown in Fig. 1(e). From the greatly reduced saturation magnetization as the *P*_O2_ increases, we can speculate the observed variation in loop shape is probably related to the reduced ferromagnetism with the formation of oxidized Co and Fe.

**Figure 2:**
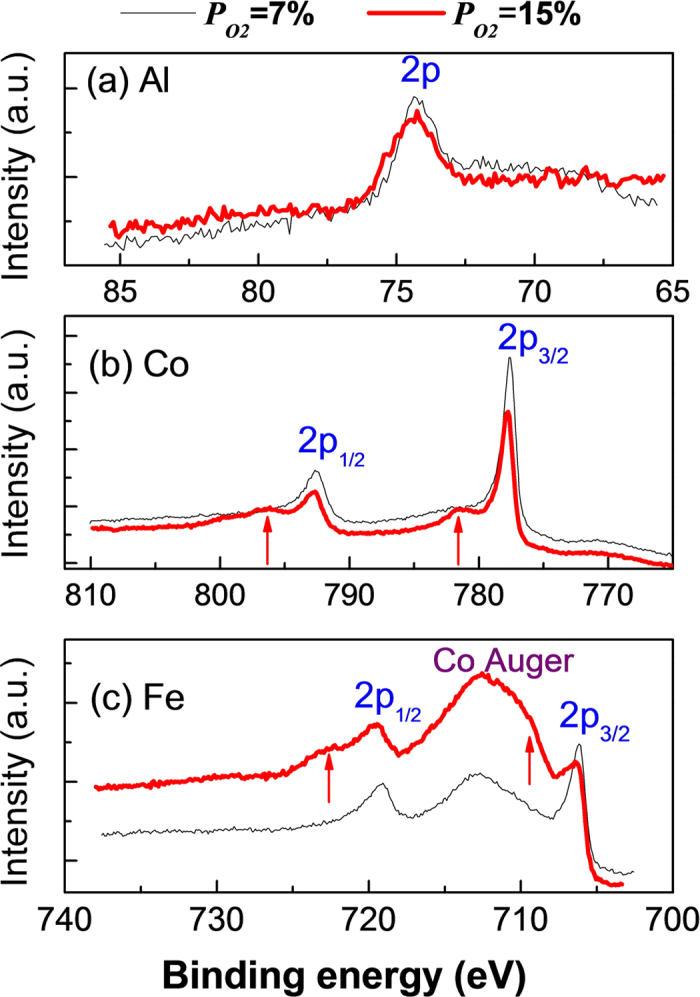
Component analyses. The XPS spectra of (**a**) Al-2p, (**b**) Fe-2p, and (**c**) Co-2p for 2.1 nm thick CFAO samples prepared at *P*_*O2*_ = 7% (black thin lines) and 15% (red thick lines). Note that the satellite peaks are indicated by arrows.

In order to clarify the oxidation effect on the crystalline structure and chemical states of the elements in the CFAO layer, x-ray diffraction and photoelectron spectroscopy (XPS) measurements were performed. We found no diffraction peaks related to Co, Fe, or CoFe alloy, implying that the CFAO layer is in amorphous or very fine nanocrystalline state. Figure 2 shows the XPS spectra for two typical samples of *P*_O2 _= 7% and 15%. The single peak observed in Fig. 2(a) at 74.4 eV corresponds to the binding energy of Al^3+^-2p, indicating that the Al was full oxidized for both samples due to the strong affinity of aluminum for oxygen. Figure 2(b) displays two sharp peaks of Co 2p_3/2_ and Co 2p_1/2_, located at 777.7 eV and 792.7 eV, respectively. However, as compared to the spectrum of *P*_O2 _= 7%, two additional satellite peaks (shown by arrows) appear for the case of *P*_O2 _= 15%, which suggests the Co was partially oxidized. In Fig. 2(c), although there exists a broad Co auger peak, the presence of a satellite peak for *P*_O2 _= 15% can also be identified from the main Fe 2p_3/2_ and 2p_1/2_ peaks. These results verify that at low *P*_O2_ the CFAO films are mainly composed of metallic CoFe particles separated by amorphous Al_2_O_3_ matrix. As the oxygen pressure increases, partial Fe and Co get oxidized. The formation of CoFe oxides should be responsible for the observed serious reduction of saturation magnetization and magnetic anisotropy at high *P*_O2_.

**Figure 3:**
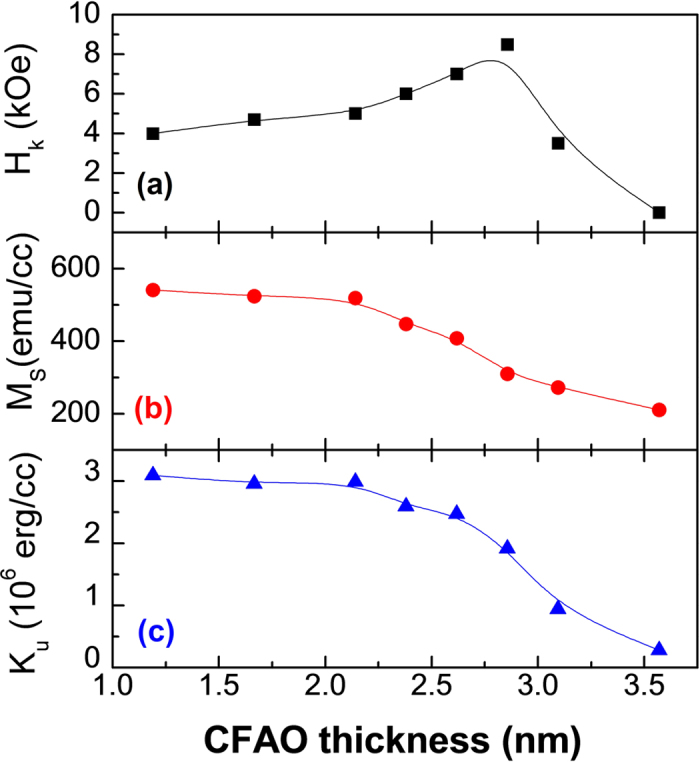
Static magnetic properties. The CFAO thickness dependence of (**a**) effective perpendicular anisotropy field *H*_*k*_, (**b**) saturation magnetization *M*_S_, and (**c**) intrinsic anisotropy energy *K*_u_ measured at room temperature, here the oxygen partial pressure is 7%.

The influence of CFAO thickness on magnetic anisotropy was also examined. Samples with different *t*_*CFAO*_ were prepared at a fixed *P*_O2_ of 7%. The effective perpendicular anisotropy field *H*_*k*_, the saturation magnetization *M*_*S*_, and the uniaxial magnetic anisotropy energy *K*_*u*_ were extracted from the magnetic hysteresis loops and plotted in Fig. 3 as a function of *t*_*CFAO*_. Similar to its dependence on *P*_O2_, the *H*_*k*_ also exhibits a non-monotonic variation behavior, reaching a maximum value about 8.0 kOe at *t*_*CFAO *_= 2.8 nm. In comparison, the *M*_*S*_ slightly decreases at *t*_*CFAO *_< 2.1 nm, above which it begins to drop dramatically. Similar to Fig. 1(e), the sample of *t*_*CFAO *_> 3.6 nm also has no magnetic anisotropy, i.e. the magnetic loops measured at any field direction are the same and show zero remanence. The *K*_*u*_ value, calculated according to the relation of 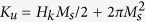, remains almost unchanged for *t*_*CFAO *_< 2.1 nm and then decreases monotonically, following a similar trend to that of *M*_*S*_. We should point out that the *K*_*u*_ value at *t*_*CFAO *_≤ 2.1 nm approaches the magnitude of perpendicular [Co/Ni]_N_ multilayers^25^, which is strong enough to maintain thermal stability of the spintronic elements. According to the XPS results, we know that the CFAO layer is composed of metallic CoFe nanoparticles dispersed in the amorphous Al_2_O_3_ matrix as the oxygen pressure is low. The sample presents a definite square magnetic hysteresis loop with out-of-plane easy axis. However, with increasing *P*_O2_ or *t*_*CFAO*_ the CoFe grains become partially oxidized, both PMA and *M*_*S*_ decrease simultaneously. In particular at *P*_O2 _> 20% or *t*_*CFAO*_ ≥3.6 nm, no matter which direction the external magnetic field is applied, the magnetic loops always show superparamagnetic characteristics of no hysteresis and moderate saturation field. From the similar variation trend of PMA and *M*_*S*_, we infer that the serious reduction of PMA occurred at thicker *t*_*CFAO*_ (also at higher *P*_O2_) should have the same origin as *M*_*S*_, being the result of the increased amount of CoFe oxides which has smaller magnetization and shows superparamagnetic behavior at RT.

**Figure 4:**
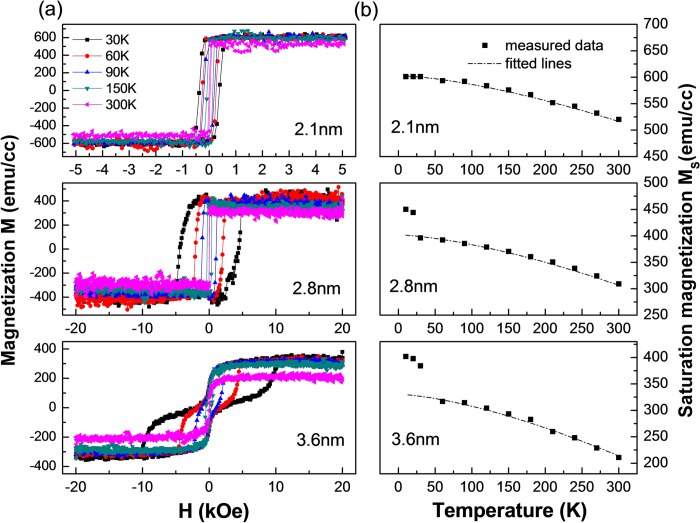
Magnetic measurements at low temperatures. (**a**) The perpendicular magnetic hysteresis loops measured by PPMS at various temperatures for samples with various CAFO thicknesses of 2.1, 2.8, and 3.6 nm. (**b**) Temperature dependence of the measured saturation magnetization *M*_S_ (solid squares) and the corresponding fitted lines (dash dot) by Bloch’s *T*^3/2^ law.

In order to further clarify this, we measured the perpendicular magnetic hysteresis loops by PPMS at reduced temperatures for the samples of *P*_O2 _= 7% with various CFAO thicknesses of 2.1, 2.8 and 3.6 nm. As shown in Fig. 4(a), these magnetic hysteresis loops vary in a distinctly different way with decreasing temperature. For the sample with a relatively thin *t*_*CFAO*_ of 2.1 nm, the perpendicular magnetic hysteresis loops exhibit a rectangle shape with a remanence ratio of 1. The *H*_c^_ value increases moderately from 18 Oe at RT to 400 Oe at 30 K due to the decreased thermal effect of the ferromagnetic CoFe alloy. However, for the thick CFAO sample of 3.6 nm, the RT magnetic loop is very slanted with no hysteresis. The slanted loop gradually transforms to a two-step switching shape at a temperature below 150 K. Note that with the decrease of measurement temperature, the first step switching is still very gradual and almost no change occurs in the switching field. In contrast, the second step switching field increases surprisingly, even reaching a value as high as 9.2 kOe at T = 30 K. We consider that the first step loop arises from the contribution of small CoFe grains while the second step from the hard magnetic phase of CoFe oxides. The CoFe oxides were reported to own larger magnetic anisotropy, but much weaker *M*_S_ and lower Curie temperature by comparison with the CoFe granules^26^,^27^. In our samples they are superparamagnetic at RT but become hard magnetic at low temperatures. Meanwhile, because of the existence of large amount of adjacent CoFe oxides in the 3.6 nm thick CFAO samples, the metallic CoFe also exhibits superparamagnetic behavior even at T = 30 K. Such two-step switching loop is just the superposition of magnetic responses from the hard magnetic CoFe oxides and the superparamagnetic metallic CoFe grains. As for the sample with an intermediate CFAO thickness of 2.8 nm, no two-step switching phenomenon takes place. The RT magnetic loop is also in a rectangle shape with a very small coercivity of 10–20 Oe, implying that the metallic CoFe grains are magnetically coupled. Therefore, at low temperatures the soft magnetic CoFe and hard magnetic CoFe oxides are rigidly exchange-coupled, behaving like a single magnet. The perpendicular coercivity *H*_c^_ increases considerably as temperature decreases, showing 4.6 kOe at T = 30 K. However, this value is much smaller than the corresponding second step switching field of the 3.6 nm thick sample. It is understandable according to the two-phase theory, since the coercivity of the exchange-coupled hard-soft composite system usually depends on the degree of the exchange coupling, and is surely lower than that of the corresponding single hard magnet^28^,^29^.

Accompanied with the changes of *H*_c^_ and loop shape, the saturation magnetization *M*_*S*_ also varies with temperature. The proportion of the hard or soft magnetic phase can be identified by fitting the temperature dependence of *M*_*S*_ shown in Fig. 4b. Clearly, for all the three samples, the *M*_*S*_ increases monotonically with the decrease of temperature. For a ferromagnetic system the saturation magnetization is known to follow the Bloch’s *T*^3/2^ law. Considering that there are two kinds of magnetic phases in our samples, we propose a function which contains two terms of Bloch’s law to simulate the curves in Fig. 4(b),
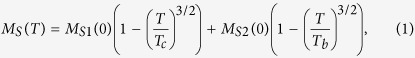


where *M*_*S*1_(0) and *M*_S2_(0) are the effective saturation magnetization of the soft and hard magnetic phases at T = 0 K, respectively. *T*_c_ denotes the Curie temperature of the soft magnetic phase while *T*_b_ refers to the blocking temperature of the hard magnetic phase. Obviously, except for the points at very low temperatures of T < 50 K, all the other data can be well fitted by equation (1). The detailed fitting parameters are listed in Table 1. The calculated *T*_*c*_ is about 1100 K, nearly equal to the Curie temperature of CoFe alloy. The *T*_b_ is as low as 310 K, being in agreement with the superparamagnetic behavior of CoFe oxides at RT. As expected, with the increase of CFAO thickness, the effective *M*_*S*1_ and *M*_*S*2_ vary in an opposite way, i.e. the proportion of the soft magnetic part decreases while that of the hard magnetic part increases. For the 2.1 nm CFAO sample, almost no hard magnetic phase exists, thus the *M*_*S*1_ of 600.6 emu/cc corresponds to the actual saturation magnetization of the soft phase. So, we can roughly estimate the ratio δ of the soft phase to the whole CFAO layer by δ = *M*_*S*1_(0)/600.6, which is calculated to be 1.0, 0.60, 0.40, and 0.16 for *t*_*CFAO *_= 2.1, 2.8, 3.6, and 4.8 nm, respectively, verifying the increased amount of hard magnetic phase with *t*_*CFAO*_. Note that for 2.8 and 3.6 nm CFAO samples, the saturation magnetization deviates away from Bloch’s law at very low temperatures, which can be attributed to the effect of some paramagnetic impurities^30^,^31^. The deviation is more seriously for 3.6 nm sample, being indicative of the presence of more impurities in the thicker CFAO layer. So in order to obtain CFAO films with strong PMA, excessive oxidation should be avoided.

**Figure 5:**
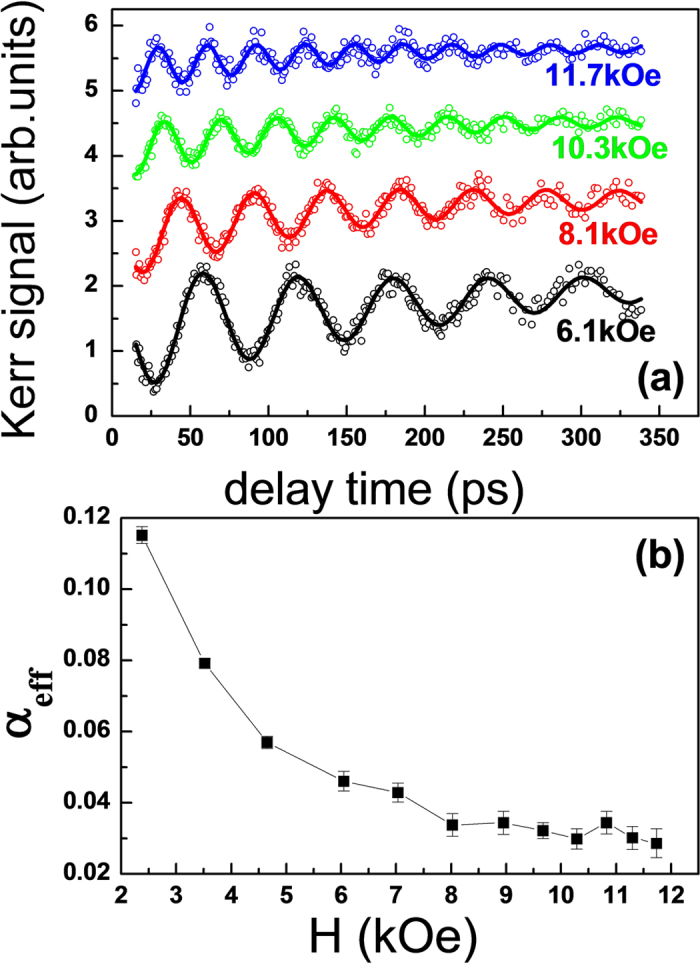
Magnetization precession and Gilbert damping. (**a**) Experimental magnetization precession and damping signals (open circles) and fitting curves (solid lines) for 2.1 nm CFAO sample at *P*_O2 _= 7%. The solid curves represent fittings with the damped sinusoidal functions. (**b**) Dependence of the effective damping parameter *α*_eff_ on external magnetic field.

In addition, laser-induced magnetization precession and damping dynamics are studied by optical pump-probe technique based on the time-resolved magneto-optical Kerr effect (TR-MOKE)^32^,^33^. Figure 5(a) shows the typical TR-MOKE curves for the CFAO sample of *t*_*CFAO*_ = 2.1 nm and *P*_O2 _= 7% under various external fields. The precession signals *θ*_*k*_ can be well fitted by an exponentially damped sinusoidal function of 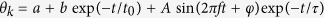
34. Here *a* is the background signal. The second term is an exponential decaying signal representing the slow recovery process, where *b* is the amplitude and *t*_0_ is the characteristic relaxation time. The third term describes the magnetization precession dynamics, the *A*, *f*, *φ*, and *τ* represent the oscillation amplitude, frequency, phase, and decay time, respectively. For the case of small damping, the effective damping parameter *α*_*eff*_ can be calculated approximately according to the relation of 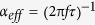 35,36. The *α*_*eff*_ contains intrinsic and extrinsic contributions. In the thinner magnetic films with PMA, the extrinsic damping mainly results from the inhomogeneous distribution of magnetization or magnetic anisotropy, which may arise from the interface roughness, thin layer thickness, and other film defects^37^,^38^,^39^. By applying a large enough magnetic field, the extrinsic damping can be well suppressed. As shown in Fig. 5(b) the obtained *α*_*eff*_ gradually decreases with increasing field, and reaches nearly a stable value of 0.028 at *H *> 10 kOe, indicating the magnetic field over 10 kOe is strong enough to eliminate the effect of local magnetic inhomogeneities^40^. Furthermore, if the spin pumping effect of the Pd underlayer is also taken into consideration^34^,^41^, the intrinsic Gilbert damping *α* should be smaller than 0.028. Compared with the damping of CFA film reported by Cui *et al.*^18^, our *α* value is relatively higher, this is because the CFAO sample has much stronger PMA strength^42^. According to these experiments, we consider that the perpendicular CFAO film has the advantage of achieving low damping for STT switching.

In summary, we have achieved strong perpendicular magnetic anisotropy in oxidized Co_50_Fe_25_Al_25_ films with a layer thickness of 1.0–3.0 nm by reactive magnetron sputtering. With increasing oxygen partial pressure or CFAO thickness, the effective perpendicular anisotropy field *H*_*k*_ shows a non-monotonic variation behavior, which initially increases and then decreases after reaching a maximum of ~8.0 kOe. Excessive oxidation will give rise to significant reduction of PMA and *M*_*S*_ due to the presence of large proportions of superparamagnetic phase and paramagnetic impurities. Such CFAO magnetic films with the advantages of small coercivity, strong PMA, proper thickness, and low damping parameter, could be used in spin valves or magnetic tunnel junctions as high performance magnetic memory elements.

## Methods

### Samples

All the samples, in a structure of Ta (3)/Pd (10)/CFAO (*t*_CFAO _= 1.2–4.8)/Ta(7) (thickness in unit of nm), were grown on Corning glass substrates by magnetron sputtering under a base pressure better than 8×10^−8^ Torr in a KJLC CMS-18 system. The bottom Ta(3)/Pd(10) layers were used as buffer layer while the top Ta (7) as capping layer. The deposition rates of Ta and Pd were 0.43 Å/s and 1.20 Å/s, respectively. The CFAO layer was formed by reactive sputtering the Co_50_Fe_25_Al_25_ target in a mixture of Ar and O_2_ gases at various oxygen partial pressures (*P*_O2_) ranging from 0 to 30%. The growth rate of metallic CFA film was 0.31 Å/s, it decreased with increasing *P*_O2_, about 0.22 Å/s at *P*_O2 _= 7% and 0.13 Å/s at *P*_O2 _= 15%.

### Static magnetic properties measurement

X-ray photoelectron spectroscopy (XPS) was used to analyze the composition and chemical state of CFAO layer. Vibrating sample magnetometer (VSM) and Physical Property Measurement System (PPMS) were used to measure the magnetic hysteresis loops at room temperature (RT) and low temperatures, respectively.

### Magnetization dynamics measurement

The time-resolved magneto-optical Kerr effect (TR-MOKE) measurements were performed at room temperature using a Ti:sapphire amplifier laser, with central wavelength of 800 nm, pulse duration of 100 fs, and repetition rate of 1 kHz. We used linearly polarized intense pump laser pulses with energy density of 0.5 mJ/cm^2^ to excite the magnetization dynamics, while much weak probe pulses of 0.06 mJ/cm^2^ to detect the pump-induced changes. The transient MOKE signal was obtained in a polar geometry, with both pump and probe pulses at almost normal incidence so that the Kerr rotation is proportional to the out-of-plane component of the magnetization. The external magnetic field was applied at an angle of ~10° with respect to film plane in order to set the magnetization orientation away from the perpendicular easy axis.

## Additional Information

**How to cite this article**: Wu, D. *et al.* Perpendicular magnetic anisotropy and magnetization dynamics in oxidized CoFeAl films. *Sci. Rep.* **5**, 12352; doi: 10.1038/srep12352 (2015).

## Figures and Tables

**Table 1:** The fitting parameters for the temperature dependence of *M*_S_ by a function including two terms of Bloch’s *T*^3/2^ law.

***t_CFAO_* [nm]**	***M_S1_*(0) [emu/cc]**	***M_S2_*(0) [emu/cc]**	***T_c_* [K]**	***T_b_* [K]**	**δ**
2.1	600.6	2.8	1151 ± 10	319 ± 8	1.0
2.8	359.2	41.6	1100 ± 12	320 ± 3	0.60
3.6	237.5	96.3	1145 ± 0.5	315 ± 5	0.40
4.8	94.1	129.9	1101 ±3 6	314 ± 1	0.16

## References

[b1] Nishimura N. *et al.* Magnetic tunnel junction device with perpendicular magnetization films for high-density magnetic random access memory. J. Appl. Phys. 91, 5246–5249 (2002)

[b2] Mangin S. *et al.* Current-induced magnetization reversal in nanopillars with perpendicular anisotropy, Nat. Mater. 5, 210–215 (2006).

[b3] Seki T. *et al.* Spin-polarized current-induced magnetization reversal in perpendicularly magnetized L1_0_-FePt layers. Appl. Phys. Lett. 88, 172504 (2006).

[b4] Yakushiji K. *et al.* Spin-transfer switching and thermal stability in an FePt/Au/FePt nanopillar prepared by alternate monatomic layer deposition. Appl. Phys. Express 1, 041302 (2008).

[b5] Meng H. & Wang J. P. Spin transfer in nanomagnetic devices with perpendicular anisotropy. Appl. Phys. Lett. 88, 172506 (2006).

[b6] Tudosa I., Katine J. A., Mangin S. & Fullerton E. E. Perpendicular spin-torque switching with a synthetic antiferromagnetic reference layer. Appl. Phys. Lett. 96, 212504 (2010).

[b7] Sim C. H., Lua S. Y. H., Liew T. & Zhu J. G. Current driven oscillation and switching in Co/Pd perpendicular giant magnetoresistance multilayer. J. Appl. Phys. 109, 07C905 (2011).

[b8] Dai B., Kato T., Iwata S. & Tsunashima S. Temperature dependence of critical current density of spin transfer torque switching amorphous GdFeCo for thermally assisted MRAM. IEEE Trans. Magn. 49, 4359–4362(2013).

[b9] Ikeda S. *et al.* A perpendicular-anisotropy CoFeB–MgO magnetic tunnel junction. Nat. Mater. 9, 721–724 (2010). 20622862 10.1038/nmat2804

[b10] Meng H., Sbiaa R., Wang C. C., Lua S. Y. H. & Akhtar M. A. K. Annealing temperature window for tunneling magnetoresistance and spin torque switching in CoFeB/MgO/CoFeB perpendicular magnetic tunnel junctions. J. Appl. Phys. 110, 103915 (2011).

[b11] Wang W. G. *et al.* Rapid thermal annealing study of magnetoresistance and perpendicular anisotropy in magnetic tunnel junctions based on MgO and CoFeB. Appl. Phys. Lett. 99, 102502 (2011).

[b12] Wang W. X. *et al.* The perpendicular anisotropy of Co_40_Fe_40_B_20_ sandwiched between Ta and MgO layers and its application in CoFeB/MgO/CoFeB tunnel junction. Appl. Phys. Lett. 99, 012502 (2011).

[b13] Sato H. *et al.* Perpendicular-anisotropy CoFeB-MgO magnetic tunnel junctions with a MgO/CoFeB/Ta/CoFeB/MgO recording structure. Appl. Phys. Lett. 101, 022414 (2012).

[b14] Yamane K. *et al.* Spin torque switching of perpendicularly magnetized CoFeB-Based tunnel junctions with high thermal tolerance. IEEE Trans. Magn. 49, 4335–4338 (2013).

[b15] Mangin S., Henry Y., Ravelosona D., Katine J. A. & Fullerton E. E. Reducing the critical current for spin-transfer switching of perpendicularly magnetized nanomagnets. Appl. Phys. Lett. 94, 012502 (2009).

[b16] Wen Z. C., Sukegawa H., Mitani S. & Inomata K. Perpendicular magnetization of Co_2_FeAl full-Heusler alloy films induced by MgO Interface. Appl. Phys. Lett. 98, 242507 (2011).

[b17] Li X. Q. *et al.* Perpendicular magnetic anisotropy of full-Heusler films in Pt/Co_2_FeAl/MgO trilayers. Appl. Phys. Express 4, 043006 (2011).

[b18] Cui Y. S. *et al.* Interfacial perpendicular magnetic anisotropy and damping parameter in ultra thin Co_2_FeAl films. Appl. Phys. Lett. 102, 162403 (2013).

[b19] Gabor M. S., Petrisor T. Jr., Tiusan C. & Petrisor T. Perpendicular magnetic anisotropy in Ta/Co_2_FeAl/MgO multilayers. J. Appl. Phys. 114, 063905 (2013).

[b20] Takamura Y., Suzuki T., Fujino Y. & Nakagawa S. Full-Heusler Co_2_FeSi alloy thin films with perpendicular magnetic anisotropy induced by MgO-interfaces. J. Appl. Phys. 115, 17C732 (2014).

[b21] Liu X., Zhang W., Carter M. J. & Xiao G. Ferromagnetic resonance and damping properties of CoFeB thin films as free layers in MgO-based magnetic tunnel junctions. J. Appl. Phys. 110, 033910(2011).

[b22] Yang H. X. *et al.* First-principles investigation of the very large perpendicular magnetic anisotropy at Fe|MgO and Co|MgO interfaces. Phys. Rev. B 84, 054401 (2011).

[b23] Nistor L. E., Rodmacq B., Auffret S. & Dieny B. Pt/Co/oxide and oxide/Co/Pt electrodes for perpendicular magnetic tunnel junctions. Appl. Phys. Lett. 94, 012512 (2009).

[b24] Monso S. *et al.* Crossover from in-plane to perpendicular anisotropy in Pt/CoFe/AlO_x_ sandwiches as a function of Al oxidation: A very accurate control of the oxidation of tunnel barriers. Appl. Phys. Lett. 80, 4157 (2002).

[b25] Wu D., Chen S. H., Zhang Z. Z., Ma B. & Jin Q. Y. Enhancement of perpendicular magnetic anisotropy in Co/Ni multilayers by *in situ* annealing the Ta/Cu under-layers. Appl. Phys. Lett. 103, 242401 (2013).

[b26] Yanagihara H., Utsumi Y., Niizeki T., Inoue J. & Kita E. Perpendicular magnetic anisotropy in epitaxially strained cobalt-ferrite (001) thin films. J. Appl. Phys. 115, 17A719 (2014).

[b27] Manova E. *et al.* Mechano-Synthesis, Characterization, and magnetic properties of nanoparticles of cobalt ferrite, CoFe_2_O_4_. Chem. Mater. 16, 5689–5696(2004).

[b28] Hernando A., Marín P., Vázquez M., Barandiarán J. M. & Herzer G. Thermal dependence of coercivity in soft magnetic nanocrystals. Phys. Rev. B 58, 366–370 (1998).

[b29] Zhao H., Li X., Zhang Z. Z., Ma B. & Jin Q. Y. Study of spin valves with *L*10-FePt pinning layer and different pinned layers. IEEE Trans. Magn. 43, 2839–2841 (2007).

[b30] Maaz K., Mumtaz A., Hasanain S. K. & Bertino M. F. Temperature dependent coercivity and magnetization of nickel ferrite nanoparticles. J. Magn. Magn. Mater. 322, 2199–2202 (2010).

[b31] Morup S. Comment on “Deviation from the Bloch T^3/2^ law in ferrite nanoparticles” by K. Mandal et al. Europhys. Lett. 77, 27003 (2007).

[b32] Van Kampen M. *et al.* All-optical probe of coherent spin waves. Phys. Rev. Lett. 88, 227201 (2002). 12059451 10.1103/PhysRevLett.88.227201

[b33] Barman A. *et al.* Time-Domain Study of Magnetization Dynamics in Magnetic Thin Films and Micro- and Nanostructures. Solid State Phys . 65, 1–108 (2014).

[b34] Mizukami S. *et al.* Gilbert damping in perpendicularly magnetized Pt/Co/Pt films investigated by all optical pump-probe technique. Appl. Phys. Lett. 96, 152502 (2010).

[b35] Malinowski G., Kuiper K. C., Lavrijsen R., Swagten H. J. M. & Koopmans B. Magnetization dynamics and Gilbert damping in ultrathin Co_48_Fe_32_B_20_ films without-of-plane anisotropy. Appl. Phys. Lett. 94, 102501 (2009).

[b36] Mills D. L. & Rezende S. M. Spin dynamics in confined magnetic structures. Vol. 2 (eds Hillebrands B. & Ounadjela K. ) Ch. 2, 27 (Springer, 2003).

[b37] Barman A. *et al.* Ultrafast magnetization dynamics in high perpendicular anisotropy [Co/Pt ]n multilayers. J. Appl. Phys. 101 , 09D102 (2007).

[b38] Schellekens A. J. *et al.* Determining the Gilbert damping in perpendicularly magnetized Pt/Co/AlOx films. Appl. Phys. Lett. 102, 082405 (2013)

[b39] Walowski J. *et al.* Intrinsic and non-local Gilbert damping in polycrystalline nickel studied by Ti: sapphire laser fs spectroscopy. J. Phys. D: Appl. Phys . 41, 164016 (2008).

[b40] Chen Z. F. *et al.* Spin waves and small intrinsic damping in an in-plane magnetized FePt film. Appl. Phys. Lett. 101, 222402 (2012)

[b41] Chen S. H. *et al.* Interfacial effect on the ferromagnetic damping of CoFeB thin films with different under-layers. Appl. Phys. Lett. 103, 032402 (2013).

[b42] Song H. S. *et al.* Relationship between Gilbert damping and magneto-crystalline anisotropy in a Ti-buffered Co/Ni multilayer system. Appl. Phys. Lett. 103, 022406 (2013).

